# Linking workplace digitalization to work engagement: evidence from China public sector organizations

**DOI:** 10.3389/fpsyg.2025.1455250

**Published:** 2025-01-29

**Authors:** Xiaohui Zhan, Shenghua Xie

**Affiliations:** ^1^International Business School, Jinan University, Zhuhai, China; ^2^School of Public Administration, Central China Normal University, Wuhan, China

**Keywords:** workplace digitalization, challenge appraisal, threat appraisal, digital literacy, work engagement, government employee

## Abstract

**Purpose:**

The construction of “Digital Government” has greatly facilitated the workplace digitalization in the public sectors of China. Workplace digitalization has become a pervasive phenomenon in modern organizations, including the public sector. Existing research on the impact of workplace digitalization on individual behavior has yielded conflicting results, making the impact of workplace digitalization on employee work engagement remains a subject of debate and investigation. Based on the transactional theory of stress, this article aims to examine how workplace digitalization influences government employees’ work engagement through different appraisals (i.e., challenge and threat) and the moderating role of a personal trait (i.e., digital literacy).

**Methods:**

Structured questionnaires and a three-wave research design were used to collect data. A total of 290 employees from public organizations in Guangdong Province, in China, participated in the study. SPSS and MPLUS were used to analyze the data using the latest bootstrapping and process macro techniques.

**Results:**

The results show that workplace digitalization can produce both beneficial and detrimental impacts on work engagement of government employees via challenge and threat appraisals, respectively. The digital literacy of government employees was confirmed to moderate the impacts of perceived workplace digitalization on stress appraisals (i.e., challenge and threat).

**Conclusion:**

Our study proposes a theoretical framework that explain the mixed impacts of workplace digitalization on government employees’ work engagement via challenge and threat appraisals. It also offers practical suggestions to public sector and managers on how to balance the challenge and threat aspects of digitalization in the workplace.

## Introduction

The extensive application of digital technologies has significantly changed the operational mode of organizations (Liu et al., 2024; Allmann and Blank, 2021). As providers of public services, governments in many countries and religions have actively engaged in promoting digital governments to meet the ever-growing public demand (Syed et al., 2023; Gil-Garcia et al., 2018; Zhou et al., 2024). Indeed, previous studies have indicated that the successful implementation of workplace digitalization (WD) in government could bring positive effect on public values, such as improving government effectiveness (Wandaogo, 2022), enhancing the quality of their service offerings (Trischler and Westman Trischler, 2022), and improving the well-being of citizens (Goedhart et al., 2022).

However, despite spending billions of dollars, the process of digitalization continues to pose challenges for many government agencies. Recent literature indicates that the failure rate of digitalization in governments of developing countries has reached 65–80% (Syed et al., 2023). Faced with such a challenging situation, many scholars have increasingly paid attention to explore the reasons behind the high failure rate of WD in government. They have found that factors such as the commitment and willingness of political leadership (Dobrolyubova et al., 2019; Dwivedi et al., 2012), as well as the lack of infrastructure (Syed et al., 2023), culture (Filatova et al., 2018), and digital capabilities (Filatova et al., 2018; Omar et al., 2017), play significant roles in the process of government digitalization. While these studies offer valuable findings, previous research has largely overlooked the significant role that government employees play in promoting the success of WD in government. It is important to emphasize that digitalization involves more than just technology (Hess et al., 2016); it also requires consideration of factors related to employees, as people play a significant role in the utilization and interaction with technology (Suchman, 2012). Some scholars have stressed that the negative attitude and lower engagement behaviors of government employees towards WD may help explain the high failure rate of digitalization in government (Syed et al., 2023; Meske and Junglas, 2021). Therefore, a key condition for the successful implementation of digital government is the high level of work engagement of public employees (Syed et al., 2023; Di Giulio and Vecchi, 2023). Given these considerations, it is crucial to explore the impact of government digitalization on the public employees’ work engagement, where refers to the extent of commitment and enthusiasm of public employees towards tasks and work related to WD.

WD primarily refers to the utilization of digital technologies in business, encompassing basic tools and some more advanced technologies (Chan et al., 2021). Unlike enterprises, the main purpose of government implementation of WD is to enhance the capacity and quality of public service provision (Di Giulio and Vecchi, 2023), which may also lead to unclear effects on public employees’ work engagement. On the one hand, the widespread application of digital technologies in the process of government digitalization may bring new job requirements to public employees, increasing their tasks of learning new knowledge and skills, as well as blurring the boundaries between work and life, thereby making individuals less engaged (Ismail, 2020; Reyt and Wiesenfeld, 2015; Mahmood et al., 2001). On the other hand, the introduction of digital technologies enables public employees to provide public services in a relatively easy and convenient manner, freeing them from tedious and repetitive administrative tasks, improving work efficiency and experience, and thus enhancing their work engagement (Meske and Junglas, 2021; Chan et al., 2021). In summary, public employees may increase their work engagement due to the benefits brought by government digitalization, but they may also reduce their engagement due to the costs associated with it.

The complex consequences of WD indicates that it may serve as a distinctive stressor for employees. Ganster and Rosen (2013) proposed that appraising stress as a challenge produces functional behavior, and appraising stress as a threat produces dysfunctional behavior. Digitalization in the workplace can result in both threatening and challenging stress behaviors, possibly due to differing subjective perceptions of stress. According to the transactional theory of stress proposed by Lazarus and Folkman (1984), a certain stressor may be simultaneously appraised as challenging or threatening and that these appraisals may provoke different coping strategies. Based on this logic, we build a contingent dual-path theoretical framework to test the double-edged effect of WD on work engagement by investigating how employees appraise digitalization. Specifically, we propose that employees who appraise WD in government as a challenge will view it as an opportunity for growth, thereby enhancing their engagement in work related to digitalization. Conversely, appraising WD in government as a threat may lead employees to perceive it as a potential risk, resulting in decreased engagement in work related to digitalization.

Additionally, it is important to explore the boundary condition that determines the extent to which public employees make a challenge or threat appraisal of government digitalization. Drawing on the transactional theory of stress (Lazarus, 1966; Lazarus and Folkman, 1984), we identify digital literacy as an important moderator. This theory proposes that personal attributes can shape stress reactions, as certain characteristics influence individual cognitive appraisal of stress (Mitchell et al., 2019; Kundi et al., 2022). Digital literacy, As a critical individual characteristic, is defined as the skills, knowledge, and abilities of a person used while engaging with digital tools (Stordy, 2015), which in turn affects how employees perceive and deal with WD. Employees with high levels of digital literacy are more likely to perceive the challenging nature of WD because they are better able to meet the requirements of digital work, whereas employees with low digital literacy are more easily to perceive WD as a threat because of their lack of digital skills and confidence in being competent in digital work (Cetindamar Kozanoglu and Abedin, 2021; Lau and Yuen, 2014). Accordingly, we propose that public workers with different levels of digital literacy are likely to develop different appraisals of WD, resulting in different work engagement.

Our study makes contributions to the body of knowledge in three ways. First, by exploring the effect of WD in the public sectors on public employees’ attitudes and behaviors, this study helps broaden existing knowledge about the individual-level consequences of WD. Previous studies have primarily focused on impacts related to utilitarian-instrumental public values (Fischer et al., 2021), such as government efficiency (Wandaogo, 2022), public service quality (Trischler and Westman Trischler, 2022), and the well-being of citizens (Goedhart et al., 2022). However, very limited studies have paid their attention to the effects of WD on public employees. Given the significant role of public employees in facilitating the success of digitalization in government, our study is the first theoretical framework to empirically examined the relationship between WD in public sectors and employee work engagement.

Second, by identifying two distinct stress appraisals (challenge and threat) that serve as mediators, our study effectively explains how digitalization in public organizations promotes or inhibits employee work engagement. Previous studies have primarily examined the mediating mechanisms through which digitalization enhances government efficiency and citizens’ well-being, focusing on variables such as collaboration between public sectors (Islam et al., 2016), data analysis and processing capabilities (Fichman et al., 2014), and transparency (Wandaogo, 2022). Among the limited empirical studies focusing on the impact of WD on government workers, Wallin et al. (2020) analyzed 101 stories collected from 81 Finnish government workers using the method of empathy-based stories (MEBS) and found that digitalization may influence employees’ attitudes and behaviors by changing work tasks and providing opportunities for job control in terms of flexibility. By examining the mediating effect of two different stress appraisals, our study offers a insightful perspective to explain the relationship between digitalization government and employees’ work engagement.

Third, by identifying employees’ digital literacy as a crucial boundary condition, our study expands the literature on WD. In the limited empirical research on the impact of WD on government employees, existing studies have primarily explored the potential moderating roles of demographic variables such as gender (Wang et al., 2020), age (Wang et al., 2020), and tenure (Tong et al., 2021) on the relationship between WD and employees’ attitudes and behaviors. While these studies help us understand the impact of demographic variables, further exploration of other key individual characteristics, especially those related to digital technology, is needed to fully analyze the effects of WD on employees. In this regard, our study contributes to the WD literature by introducing public employees’ digital literacy as a key boundary condition for the relationship between WD in government and employees’ work engagement behaviors.

**Figure 1:**
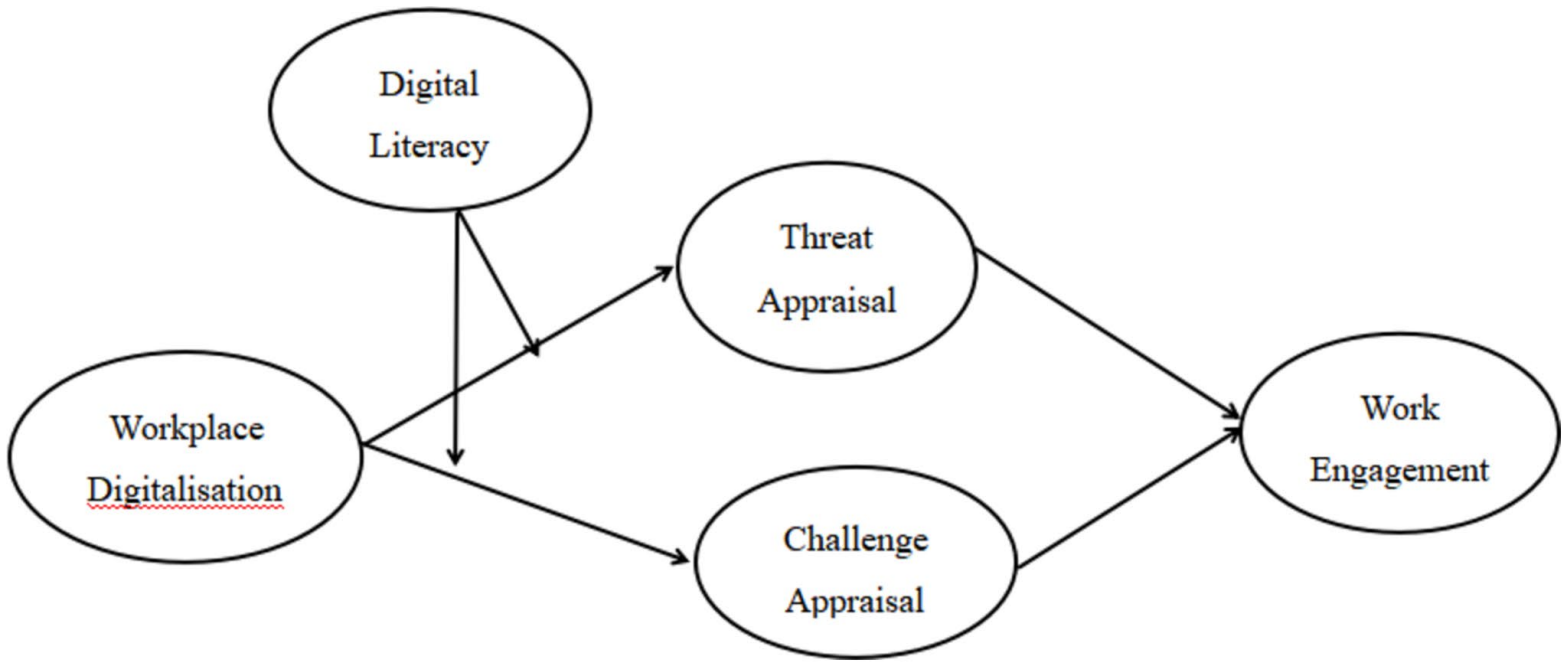
Research model.

Figure 1 illustrates the theoretical framework that we hypothesize.

## Theoretical development and hypotheses

### Workplace digitalization, threat appraisal, and work engagement

WD is an individual’s subjective perception of the application of digital technologies in business processes (Chan et al., 2021; Dziubek et al., 2022). These digitalization tools include basic technologies and some more advanced digital tools (Fischer and Pöhler, 2018). Some scholars argue that digitalization in the workplace has produced new job demands for low-skilled employees, which often exceed their existing resource levels (Cavanaugh et al., 2000; William and Cooper, 2002). WD often requires employees to take on new responsibilities, such as applying digital technologies in routine work to achieve better performance, which may make employees feel threatened and perceive it as a new stressor (Chan et al., 2021; Lazarus and Folkman, 1984). Thus, WD can represent a significant source of stress for employees.

Practitioners and scholars alike have reported inclusive findings when examining the consequences of WD. Research on WD has revealed that it serves as a double-edged sword in that it can yield mixed results, with both positive and negative impacts (Liu et al., 2024; Zhou et al., 2024; Chan et al., 2021). The cognitive appraisal theory of stress (Lazarus and Folkman, 1984) posits that employees’ cognitive appraisals of a stressor (i.e., threat and challenge appraisals) can significantly influence their responses and subsequent coping strategies towards stress (Tomaka et al., 1993). Ohly and Fritz (2010) argued that employees with threat appraisals perceive the loss and possibility of failing to perform assigned goals and tend to engage in dysfunctional behavior, whereas employees with challenge appraisals perceive the possibility of gaining from achieving certain aims and displaying functional behavior. Given that prior research has shown that WD produces both positive and negative consequences (Liu et al., 2024; Zhou et al., 2024; Chan et al., 2021), we argue that these paradoxical results can be explained by how WD is appraised.

WD, perceived as a threat, could be harmful. Employees experiencing high WD may experience psychological insecurity due to the application of new digital technologies (Liu et al., 2022). Chan et al. (2021) argued that digital presenteeism in the workplace blurs the boundary between employees’ work and social lives, resulting in long working hours. Appraising WD as a threat may make employees feel stressed and concentrate on the potential barriers of applying new technologies to assigned tasks. These experience could also bring unfavourable outcomes for employees (Karimi and Walter, 2015; Yassaee and Mettler, 2019). However, to our knowledge, there exists a dearth of empirical research examining the potential influence of WD on employee work engagement through threat appraisal. WD, appraised as a job demand (Chan et al., 2021), may deplete employees’ energy, increase stress and burnout, and decrease engagement. Therefore, based on the foregoing analyses, we inferred that threat appraisal would mediate the relationship between WD and work engagement.

*H1. Threat appraisals negatively mediate the relationship between WD and work engagement.*

### Workplace digitalization, challenge appraisal, and work engagement

WD may also be considered challenging. Highly digitized workplace can establish a foundation for employee growth and development. Lilja (2020) argued that WD could transform job design and work routines, thereby relieving employees from work tasks that repetitive, mundane and hazardous. Morris and Venkatesh (2010) suggest that digital technologies in the workplace may provide an opportunity for employees to restructure their abilities. Moreover, some studies have conclusively shown that challenging appraisal is positively associated with favorable outcomes. Ohly and Fritz (2010) found that challenge appraisals of employees is positively related to creativity and proactive behavior, which is consistent with the findings of Liu et al. (2022). O’Connor et al. (2010) empirically confirmed the positive relationship between challenge appraisal and work performance through two experimental studies.

Previous research indicates that a certain level of work stress that employees can address is expected to require more effort, thereby increasing work engagement (Petrou et al., 2017). Chan et al. (2021) argued that digital technologies in the workplace could be considered as a job resource that motivates employees to direct their energy and cognitive resources towards work, thus positively enhancing employee engagement (Pattnaik and Panda, 2020). This statement prompts us to expect increased levels of work engagement when WD is viewed as a challenge. Based on the preceding reasoning, it can be inferred that the challenge appraisal would mediated the relationship between WD and work engagement of government employees.

*H2. Challenge appraisals positively mediate the relationship between WD and work engagement.*

### The moderating influence of digital literacy

As new technologies always alter the way in which people take advantage of technologies and accomplish tasks, the definition of digital literacy changes continuously. According to Reddy et al. (2020), digital literacy was defined as the capacity of an individual to discover and effectively utilize information, create new content using this information, share and communicate this newly created information using appropriate digital technologies. Most scholars agreed that digital literacy was a multidimensional concept encompassing a range of “literacy’s” (Covello, 2010). Research on digital literacy has found that it has direct relationships with employee attitudes and behaviors, such as employability (Bejaković and Mrnjavac, 2020), work engagement (Statti and Torres, 2020) and performance (Marsh, 2018; Mohammadyari and Singh, 2015).

Lazarus’s (1966) transactional model of stress posits that specific traits can affect the stress experience of employees, as individual differences can change employees’ cognitive appraisal of stressor. In line with Lazarus’s arguments, we suggest that individual characteristics may encourage employees to focus their attention on certain aspects of a stressor. This focus can alter the way in which the stressor is appraised, which then influences the WD experience. Past studies have pinpointed digital literacy as a particularly influential personal characteristic moderating employees’ acceptance of digitalization (Hwang et al., 2016; Lei et al., 2024). As a collection of skills and abilities that related to digital technologies (Stordy, 2015), digital literacy can enable employees to perform assigned tasks effectively in a digital workplace (Reddy et al., 2020; Nawaz and Kundi, 2010). We focused on digital literacy as a moderator between workplace digitalization and stress appraisals (i.e., challenge or threat appraisals) because digital literacy enables employees to cope with WD stress more effectively (Chan et al., 2021).

Accordingly, the study proposes that digital literacy plays a moderating role in the linkage between WD and stress appraisal (i.e., challenge and threat appraisals). We further propose that low digital literacy decreases employees’ ability to overlook the negative facets of WD, such as risking losing their jobs and creating pressure to learn digital technologies, thus increasing the propensity for employees to perceive WD as a threat. On the contrary, high digital literacy can enhance employees’ positive perception of WD (e.g., opportunities to be involved in tasks that require creativity, analytical, and decision-making skills) and helps them in reframing negative stress experiences in a positive light, thus making employees more likely to appraise WD as a challenge. Therefore, we propose:

*H3a. Digital literacy moderates the positive relationship between WD and threat appraisals, such that this relationship becomes stronger when digital literacy is lower rather than higher.*

*H3b. Digital literacy moderates the positive relationship between WD and challenge appraisals, such that this relationship becomes stronger when digital literacy is higher rather than lower.*

Furthermore, digital literacy, which is crucial for surviving in the digital era (Nawaz and Kundi, 2010), can bolster employees’ motivation to utilize digital devices, platforms, and tools for their routine work. In this way, it may optimize the functions of digital technologies for work-related information support, which acts as a proximal antecedent of work engagement (Chan et al., 2021). Therefore, we predicted that digital literacy would switch the indirect relationships of WD on work engagement through threat and challenge appraisals. Specifically, employees with higher levels of digital literacy will appraise WD as a challenge rather than a threat because digital literacy enables employees to use digital tools to solve problems and perform tasks effectively in stressful situations (Nawaz and Kundi, 2010), which would ultimately enhance their work engagement. By contrast, employees with lower levels of digital literacy will perceive WD as a threat rather than a challenge because they are less competent in handling digital technologies in the workplace (Chan et al., 2021), which ultimately decreasing their work engagement. Hence, we propose:

*H4a. The indirect effect of WD on work engagement through threat appraisal will be stronger when digital literacy is lower rather than higher.*

*H4b. The indirect effect of WD on work engagement through challenge appraisal will be stronger when digital literacy is higher rather than lower.*

## Materials and methods

### Participants and procedure

Employees from four public organizations in Guangdong Province, China, participated in this survey. China is considered a suitable country for this study, as it has become one of the leading nations in digitalization in recent years, particularly in digital skills and public services, following the rapid development of its digital economy. Guangdong Province, as one of the most economically developed regions in China, is representative and forward-looking in terms of digital government construction and the digitalization of public organizations. These public workers from four public organizations were targeted because they provide essential public services, including education, health, and social security, they represent a group of employees currently working in a highly digitalized environment, and digitalization has significantly influenced their job content and methods.

The managers of these public sectors were contacted to obtain their approval to invite their employees to participate in the research project. The participants were informed about the overarching aim of our study, how it would be conducted (i.e., through three phase), and offered a guarantee of the strict confidentiality of their responses. Due to the three-wave research design of our study, the managers of four public organizations were asked to provide a list of employees who would be involved in this study, code them, and use them as clues to match the three-wave data. We collected data in three waves separated by 1 month (Matthews et al., 2014) to reduce common method variance (CMV) bias. In the first phase, scales on demographic variables, WD, and digital literacy were completed by participants. In the second phase, the survey included scales of challenge and threat appraisal. We asked participants to report on work engagement in the third phase of this survey. In the first wave, a total of 512 participants completed the survey. In the second wave, the number of respondents dwindled to 345 who finished the scales. Subsequently, 290 individuals participated in the survey in the third wave. The final sample comprised 119 women (41%), and 171 men (59%). The average age of the participants was 34.8 years (*SD* = 10.5). Regarding their tenure, the average length of service was 3.88 years (*SD* = 9.2). Of the respondents, 94.7% of all participants held a bachelor’s degree or higher.

### Measures

Based on Brislin’s (1970) back-translation procedure, the scales were translated from English to Chinese. Participants rated each item on a 5-point Likert scale ranging from 1 (indicating strongly disagree) to 5 (indicating strongly agree).

#### Workplace digitalization

WD was assessed using a 5-item scale proposed by Li (2020), also used by Li and Fei (2023). An example item was “I work in an organization that promotes digital design, manufacturing, and management (*α* = 0.806).”

#### Threat appraisal

Threat appraisal was rated on a 4-item scale developed by Drach-Zahavy and Erez (2002). An example item was “I’m worried that the task might reveal my weaknesses (α = 0.810).”

#### Challenge appraisal

We measured challenge appraisal by using a 4-item scale borrowed from Drach-Zahavy and Erez (2002). An Example item was “The task provides opportunities to strengthen my self-esteem (α = 0.806).”

#### Work engagement

We measured work engagement by using a 5-item questionnaire borrowed from Bledow et al. (2011). An example items was “I am enthusiastic about my job (α = 0.906).”

#### Digital literacy

We measured digital literacy using the 9-item scale developed by Lau and Yuen (2014), which was also used by Alma Çallı et al. (2022). According to Lau and Yuen (2014), digital literacy has three aspects: information, the internet, and computer literacy. A Sample item was “I can judge the degree to which information is practical or satisfies the needs of the task, including determining authority, bias, and timeliness of materials (α = 0.914).”

#### Control variables

In alignment with prior studies (Bakker et al., 2005; Kundi et al., 2022), gender, age, education level, and organizational tenure were selected as control variables.

### Data analysis

Descriptive statistics and correlation analysis were performed using SPSS version 20.0, with *p* < 0.05 (two-tailed test) considered statistically significant. Pearson’s correlation analysis was used to examine the relationships between WD, threat appraisal, challenge appraisal, digital literacy, and work engagement. Structural equation modeling was conducted using MPLUS version 7.0 to test the discriminant validity of the main variables, as well as the mediating effects of threat and challenge appraisals. The moderating effect of digital literacy and the moderated mediation effects were analyzed using SPSS and PROCESS version 3.4 Model 7, respectively.

## Results

### Descriptive statistics and correlation analysis

Table 1 presents the means, standard deviations, and intercorrelations among the study variables.

**Table 1:** Descriptive analysis and correlations.

Variables	1	2	3	4	5	6	7	8	9
1. Gender									
2. Age	0.095								
3. Education	0.048	−0.033							
4. Tenure	0.146*	0.144*	0.144*						
5. WD	−0.368**	0.002	−0.115*	−0.097					
6. TA	−0.438**	−0.310**	−0.132	−0.314	0.301***				
7. CA	−0.093	0.206	0.121	0.228*	0.326***	−0.198**			
8. DL	−0.078	0.096	0.002	0.016	0.220***	−0.093	0.205***		
9. WE	0.007	0.289	0.044	0.149*	0.045	−0.307***	0.370***	0.161**	
Mean	1.41	34.8	2.45	3.88	2.881	2.969	2.698	3.134	3.669
SD	0.493	10.5	0.753	9.2	0.868	0.855	0.707	0.881	0.932

*N* = 290; *p < 0.05,**p < 0.01, ***p < 0.001; WD = workplace digitalization; TA = threat appraisal; CA = challenge appraisal; DL = digital literacy; WE = work engagement.

### Measurement model analysis

Confirmatory factor analysis (CFA) was performed using MPLUS software to test the distinctiveness of the five variables included in this study: WD, challenge appraisal, threat appraisal, digital literacy, and work engagement. As shown in Table 2, compared with several alternative models, the hypothesized five-factor model produced an extremely good fit to the data (*χ*^2^ = 256.805, df = 160, CFI = 0.948, TLI = 0.939, RMSEA = 0.056, and SRMR = 0.056), which provided enough evidence to support the distinctive nature of our study variables. Moreover, the presence of a common method effect should be examined because all variables were collected from the same source (i.e., employees). According to Kundi et al. (2021)´s suggestion, a CFA version of Harman’s single-factor test was conducted, and the results indicated that the single-factor model exhibited a poor fit to the data (*χ*^2^ = 3337.224, df = 560, CFI = 0.450, TLI = 0.416, RMSEA = 0.135, and SRMR = 0.159). These results provide empirical evidence that common method bias is unlikely to be a problem in our study.

**Table 2:** Structural validity.

	*χ* ^2^	df	CFI	TLI	RMSEA	SRMR
Model 1 (hypothesized five-factor model)	256.805	190	0.948	0.939	0.056	0.056
Model 2 (four-factor model: combines TA and CA)	427.447	164	0.860	0.837	0.083	0.091
Model 3 (four-factor model: combines WD and DL)	397.595	164	0.876	0.856	0.079	0.071
Model 4 (one-factor model)	3337.224	560	0.450	0.416	0.135	0.159

WD = workplace digitalization; TA = threat appraisal; CA = challenge appraisal; DL = digital literacy; WE = work engagement.

### Hypotheses testing

**Figure 2:**
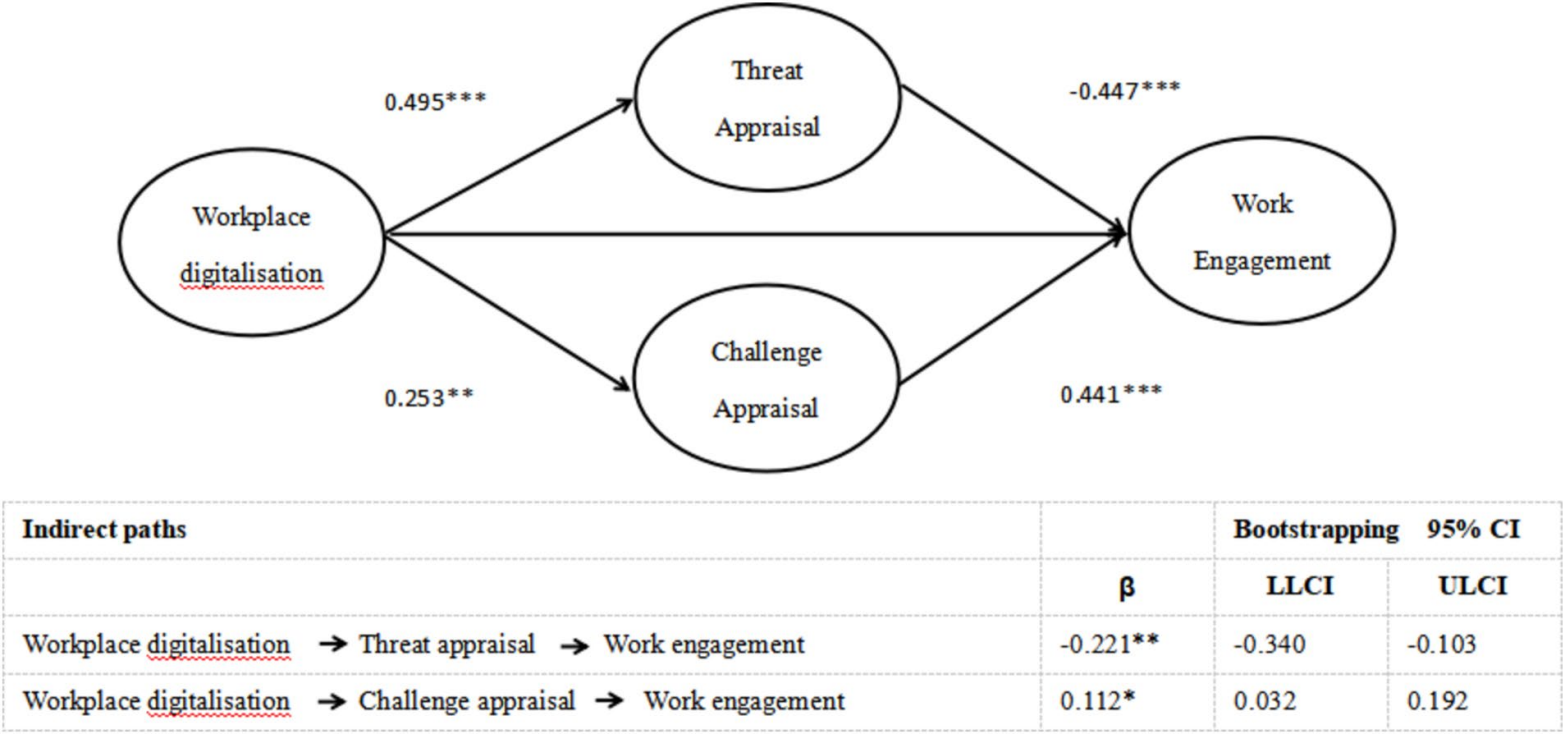
Results of structural model (*N* = 290; *p < 0.05,**p < 0.01, ***p < 0.001).

To test the mediating roles of threat and challenge appraisals on WD and work engagement, we employed MPLUS software to build a structural equation model, which produced an acceptable goodness of fit (*χ*^2^ = 276.603, df = 130, CFI = 0.911, TLI = 0.905, RMSEA = 0.073, and SRMR = 0.073). Figure 2 shows the results of mediating tests. As the indirect impacts of WD on work engagement via threat appraisal was significant and negative (β = −0.221, 95% CI = [−0.340, −0.103]), threat appraisal played a significant mediating role in the relationship between WD and work engagement, supporting Hypothesis 1. Moreover, the indirect impact of WD on work engagement through challenge appraisal was significant and positive (β = 0.112, 95% CI = [0.032, 0.192]). Thus, challenge appraisal significantly mediated the influence of WD on work engagement, supporting Hypothesis 2.

**Figure 3:**
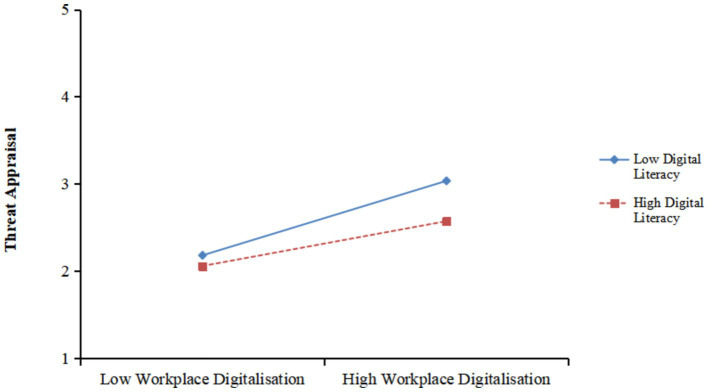
Interaction between workplace digitalisation and digital literacy on threat appraisal.

Next, we tested the moderation role of digital literacy on the relationship between WD and appraisals using SPSS (20th edition) and PROCESS (Model 7). As shown in Table 3, the interaction between WD and digital literacy had a significant and negative effect on threat appraisal (β = −0.185, *p* < 0.01). It can be seen from Figure 3 that the effect of WD on threat appraisal was stronger when digital literacy is lower than higher. Therefore, Hypothesis 3a was supported.

**Table 3:** The moderating effect test of digital literacy.

	Challenge appraisal		Threat appraisal	
	β	SE	β	SE
Workplace digitalization	0.221***	0.044	0.342***	0.056
Digital literacy	0.180**	0.044	−0.146**	0.055
Workplace digitalization × Digital literacy	0.175***	0.031	−0.185**	0.040

*N* = 290; *p < 0.05, **p < 0.01, ***p < 0.001.

**Figure 4:**
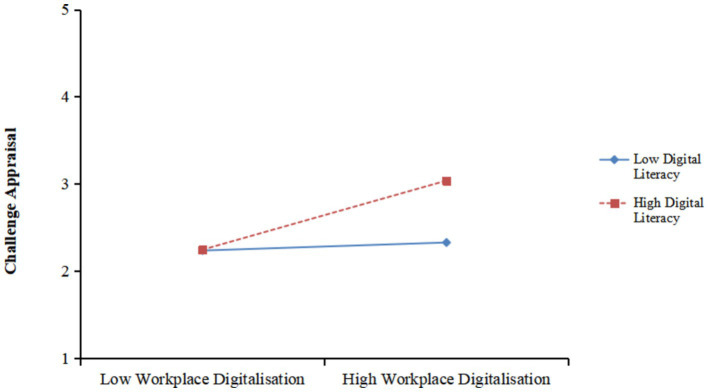
Interaction between workplace digitalisation and digital literacy on challenge appraisal.

Furthermore, in Table 3, the interaction between WD and digital literacy had a significant positive effect on challenge appraisal (β = 0.175, *p* < 0.001). Figure 4 shows the effect of perceived WD on challenge appraisal was stronger for high digital literacy employees than low digital literacy ones. Therefore, Hypothesis 3b was supported.

Table 4 lists the results of moderation mediation effect tests. The indirect effect of WD on work engagement through threat appraisal was stronger for high digital literacy employees (β = −0.066, 95% CI = [−0.141, 0.020]) than low digital literacy ones (β = −0.119, 95% CI = [−0.211, −0.056]). Thus, hypotheses 4a was supported. Furthermore, as depicted in Table 4, high digital literacy employees’ perceived WD had stronger indirect effect through challenge appraisal on work engagement (β = 0.175, 95% CI = [0.106, 0.264]) than those of low digital literacy employees (β = 0.007, 95% CI = [−0.038, 0.063]). Therefore, hypotheses 4b was supported.

**Table 4:** The test of moderation mediation effect at different levels of digital literacy.

Work engagement	Effect	Boot SE	LLCI	ULCI
Conditional indirect effect through challenge appraisal				
Digital literacy (−1SD)	0.007	0.024	−0.038	0.063
Digital literacy (*M*)	0.091	0.025	0.048	0.150
Digital literacy (+1SD)	0.175	0.040	0.106	0.264
Conditional indirect effect through threat appraisal				
Digital literacy (−1SD)	−0.119	0.038	−0.211	−0.056
Digital literacy (*M*)	−0.093	0.030	−0.168	−0.045
Digital literacy (+1SD)	−0.066	0.029	−0.141	0.020

*N = 290; M, mean; SD, standard deviation.*

## Discussion

### Theoretical implications

Our study offers several theoretical implications. First, by taking WD as a whole, this study explores the impact of WD in government on employee-level factors, specifically work engagement, thereby expanding the literature on WD in the field of organizational management. In recent years, an increasing number of public agencies have chosen to undergo digitalization to achieve public values (Fischer et al., 2021). Scholars’ interest in government digitalization has also been growing (Liu et al., 2024; Fischer et al., 2021). However, existing research on government digitalization primarily focuses on its macro-level outcomes, such as uch as government efficiency (Wandaogo, 2022), public service quality (Trischler and Westman Trischler, 2022), and the well-being of citizens (Goedhart et al., 2022). Few studies have examined the effects of WD on individual-level factors. In response to the call by Liu et al. (2024), this study links WD to work engagement of government employees, thus enriching the literature on the nomological network of WD consequences.

Second, by introducing the mediating effects of different cognitive mechanisms (challenge and threat), our research provides a new approach to explain the relationship between WD in government and work engagement of public employees. Existing studies on the mediating variables of WD in government are primarily divided into two perspectives: one perspective focuses on macro-level variables, suggesting that digitalization enhances government efficiency by improving collaboration between public sectors (Islam et al., 2016), data analysis and processing capabilities (Fichman et al., 2014), and transparency (Wandaogo, 2022); the other perspective focuses on public psychological variables, proposing that government digitalization boosts citizens’ well-being by increasing public participation and enhancing trust (Afiyah, 2024; Virnandes et al., 2024). Among the limited studies focusing on the impact of government digitalization on employees, Wallin et al. (2020) collected data from 81 Finnish government workers using the method of empathy-based stories (MEBS) and found that digitalization may influence employees’ attitudes and behaviors by changing work tasks and providing opportunities for job control in terms of flexibility. However, less attention has been paid to the public employees’ appraisal of digitalization in government. Based on the transactional theory of stress (1984), we argue that government digitalization could perceived as a workplace stressor, to which public employees make challenge or threat appraisals. These appraisals, in turn, lead to subsequent increases or decreases in their work engagement. By demonstrating that WD can either enhance or inhibit work engagement through challenge and threat appraisals, our study makes its second contribution to the literature on WD, providing a novel perspective to explain the relationship between WD and work engagement of public workers.

Third, by identifying key factor that moderate the relationship between WD and work engagement, our study contributes to the development of the literature on WD in government. Previous limited studies have primarily explored the potential moderating effects of demographic variables, such as age (Wang et al., 2020), gender (Wang et al., 2020), and organizational tenure (Tong et al., 2021), on employees’ attitudes and behaviors towards WD, while neglecting other aspects of employee characteristics. Meanwhile, prior research has indicated gaps in understanding the moderating factors of employees’ attitudes and behaviors towards digitalization (Meske and Junglas, 2021; Chan et al., 2021; Bala and Venkatesh, 2016). Drawing on the transactional theory of stress, our study finds that digital literacy can serve as a boundary condition, moderating the impact of WD on appraisals and outcomes. In doing so, our study advances the literature on WD by introducing digital literacy as an important individual characteristic to explore the contingency for the relationship between digitalization and government employees’ attitudes and behaviors.

### Practical implications

This study also provides insights into management practices related to WD. First, the findings of this study reveal that WD has a double-edged sword effect on government employees’ work engagement. As previously mentioned, many public employees struggle to adapt to the challenges brought about by WD (Blanchard, 2018). Therefore, it is essential for managers of public sectors to closely monitor employees’ attitudes towards WD and take supportive measures to help employees better adapt to the changes in the work environment due to digitalization in government. At the same time, managers should also provide employees with necessary information related to WD, such as the goal, content, and processes involved, to increase employees’ acceptance and adaptability to WD, thereby promoting their engagement at work.

Second, this study also explores the impact mechanism of WD on employee engagement through a dual-path model and examines the mediating role of challenge and threat appraisals. Employees who perceive WD as challenging or beneficial may be motivated to engage in positive behaviors, while those who view WD as threatening or harmful may exhibit negative behaviors at work. Therefore, public organization managers should consider how they communicate policies regarding WD to their employees to reduce the likelihood of these policies being negatively appraised. One possible approach is to emphasize the benefits and opportunities of WD to employees (such as increased work efficiency and autonomy), helping them form a positive evaluation of WD, which contributes to improving employee engagement.

Finally, this study discovered that high digital-literacy employees easily focused the challenging facets of WD while seemingly ignoring its threatening aspects. They were found to have greater work engagement behavior owing to WD. However, employees with low digital literacy are more easily to perceive digitalization as a threat, ultimately lowering their work engagement. Previous research has demonstrated that certain characteristics produce positive motivational states and behaviors (Morgeson et al., 2007; Zeigler-Hill et al., 2019). Therefore, an increasing number of public organizations use personality measures to assess important attributes in their hiring and selecting processes. In this regard, our findings suggest that screening for digital literacy is valuable, especially for those who experience WD. Moreover, public organizations can enhance the perceived value of digital tools by integrating basic technologies such as social media, mobile devices, or the Internet of Things, into the workplace, enabling employees to become adept with these digital tools through regular use. Finally, interventions such as training or coaching sessions on learning new technologies should be arranged to improve government employees’ digital literacy.

### Limitations and future research

Several limitations of our study should be mentioned. First, all variables in this study were collected data from employees’ self-reported, which may raise concerns regarding common method variance (CMV) bias (Podsakoff et al., 2012). In our surveys, several remedies were used to address the possibility of CMV. First, employees were asked to report on scales of independent variable, mediation variable and dependent variable at three different time points. Second, a multilevel confirmatory factor analysis (CFA) was conducted and demonstrated that CMV was unlikely to be a serious threat. Third, our theoretical model included a moderated variable that previous studies have suggested cannot be inflated by the CMV (Podsakoff et al., 2012; Siemsen et al., 2010). The current study has confirmed the significant moderating effect of digital literacy in the relationship between WD and work engagement (via challenge and threat appraisals). Thus, according to Podsakoff et al. (2012), CMV is unlikely to present bias. However, experimental or longitudinal research designs should be adopted in future research to mitigate the CMV and strengthen the assumptions of causality among the variables.

Another possible limitation concerns the generalizability of our findings to other countries and regions, as our sample was from China. Future research could test our model in other contexts and across a range of cultures. Finally, our study suggested that digital literacy plays a crucial role in weakening the linkage between WD and threat appraisal. Future research may benefit from examining other personality characteristics (such as a promotion versus prevention focus) and contextual factors (such as organizational support in a digitalized workplace) within our theoretical framework.

## Conclusion

Nowadays, government digitalization has become an irreversible global trend, bringing pressure and challenges to public employees. Most existing research focuses on the macro-level impacts of WD in organizational management, with less attention given to its effects on employee-level factors. To address this gap, our study, based on the transactional theory of stress, identifies challenge and threat appraisals as dual mediating mechanisms and reveals digital literacy as an important boundary condition, exploring the impact mechanism of WD on work engagement of government employees. Our research provides theoretical and practical guidance for public sectors to successfully implement digitalization and help public employees adapt to it.

## Data Availability

The raw data supporting the conclusions of this article will be made available by the authors, without undue reservation.
